# Cognition in Chiari Malformation Type I: an Update of a Systematic Review

**DOI:** 10.1007/s11065-023-09622-2

**Published:** 2023-10-06

**Authors:** Maitane García, Imanol Amayra, Manuel Pérez, Monika Salgueiro, Oscar Martínez, Juan Francisco López-Paz, Philip A. Allen

**Affiliations:** 1 Department of Psychology, Faculty of Health Sciences, Neuro-E-Motion Research Team, University of Deusto, Bilbao, Spain; 2 Faculty of Health Sciences, Isabel I University, Burgos, Spain; 3 Department of Clinical and Health Psychology, and Research Methodology, Faculty of Psychology, University of the Basque Country, Donostia, Spain; 4 Conquer Chiari Research Center, Department of Psychology, University of Akron, Akron, OH USA

**Keywords:** Chiari malformation type I, Cognition, Cerebellum, Systematic review

## Abstract

Chiari malformation has been classified as a group of posterior cranial fossa disorders characterized by hindbrain herniation. Chiari malformation type I (CM-I) is the most common subtype, ranging from asymptomatic patients to those with severe disorders. Research about clinical manifestations or medical treatments is still growing, but cognitive functioning has been less explored. The aim of this systematic review is to update the literature search about cognitive deficits in CM-I patients. A literature search was performed through the following electronic databases: MEDLINE, PsychINFO, Pubmed, Cochrane Library, Scopus, and Web of Science. The date last searched was February 1, 2023. The inclusion criteria were as follows: (a) include pediatric or adult participants with a CM-I diagnosis, (b) include cognitive or neuropsychological assessment with standardized tests, (c) be published in English or Spanish, and (d) be empirical studies. Articles that did not report empirical data, textbooks and conference abstracts were excluded. After the screening, twenty-eight articles were included in this systematic review. From those, twenty-one articles were focused on adult samples and seven included pediatric patients. There is a great heterogeneity in the recruited samples, followed methodology and administered neurocognitive protocols. Cognitive functioning appears to be affected in CM-I patients, at least some aspects of attention, executive functions, visuospatial abilities, episodic memory, or processing speed. However, these results require careful interpretation due to the methodological limitations of the studies. Although it is difficult to draw a clear profile of cognitive deficits related to CM-I, the literature suggests that cognitive dysfunction may be a symptom of CM-I. This suggest that clinicians should include cognitive assessment in their diagnostic procedures used for CM-I. In summary, further research is needed to determine a well-defined cognitive profile related to CM-I, favoring a multidisciplinary approach of this disorder.

## Introduction

Chiari malformations (CM) are a group of posterior cranial fossa disorders characterized by hindbrain herniation (Manto & Christian, 2013; Tubbs et al., 2020). From the first description of the different subtypes by Hans von Chiari at the end of nineteenth century, the current classification includes six main typologies (0, I, 1.5, II, III, and IV) based upon their pathophysiology and clinical presentation, in addition to two more recently proposed categories (3.5 and V) (Massimi et al., 2011; Rindler & Chern, 2020; Tubbs & Turgut, 2020) (see Table 1 for a more detailed description). Accurate data on the prevalence of each CM typology are not available, but it is known that Chiari malformation type I (CM-I) is the most frequent, with estimated rates around 1/1000–5000 cases (Urbizu et al., 2013), although a higher prevalence has been reported in retrospective magnetic resonance imaging (MRI) studies with general population (Öktem et al., 2016; Smith et al., 2013; Vernooij et al., 2007).

**Table 1:** Classification of Chiari malformations (Tubbs et al., 2020)

**Chiari malformation subtype**	**Anatomical signs**	**Clinical presentation**
Type 0	• No hindrain herniation or minimal (< 3 mm) • PCF volume anomalies • Syringomyelia	• Chiari-like headaches related to pressure changes (short duration) • Limb weakness, scoliosis and paresthesias
Type I	• Tonsillar herniation (> 3–5 mm), usually asymmetric position • PCF volume anomalies • Syringomyelia • Hydrocephalus (less frequent)	• Headache • Neck pain, limb weakness
Type 1.5	• Tonsillar herniation • Elongation and inferior displacement of the brainstem and obex (less frequent)	• Headache • Other nonspecific symptoms (i.e., shortness of breath, difficulty speaking, lethargy)
Type II	• Tonsillar ectopia • Myelomeningocele • Elongation and inferior displacement of cerebellar vermis, brainstem and fourth ventricle • PCF volume anomalies accompanied of a set of cranial and spinal malformations • Syringomyelia • Hydrocephalus	• Brainstem symptoms • Cranial nerves anomalies-related symptoms (glossopharyngeal and vagus) • Ophthalmologic symptoms (i.e., nystagmus, abnormal pursuit movements)
Type III	• Severe hindbrain deformation and abnormal midbrain • Occipital encephalocele and osseous anomalies • Cerebellar atrophy and tethered cord • Syringomyelia • Hydrocephalus	• Severe neurological deficits and developmental disorders • High mortality rates
Type IV	• Occipital encephalocele with supratentorial contents • Significant hypoplasia and cerebellar atrophy • PCF volume abnormalities • Hydrocephalus	• Not compatible with life

There has been also reported two more subtypes in case studies:

“Chiari 3.5 malformation”: encephalomyelocystocele, characterized by occipitocervical encephalocele, and PCF anatomical abnormalities

“Chiari V malformation”: absence of the cerebellum and occipital lobes herniation through the foramen magnum

**Fig. 1:**
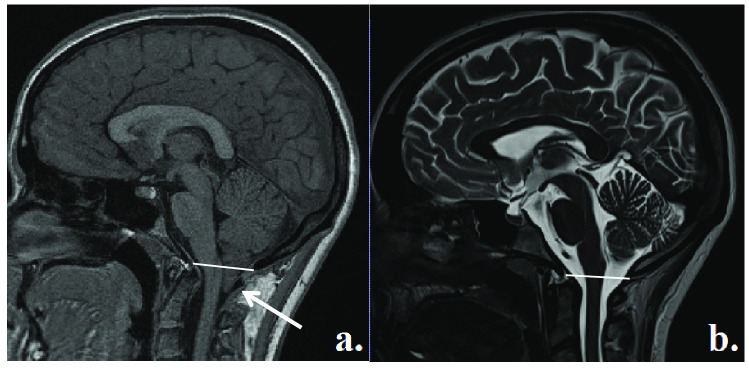
**a** T1-weighted mid-sagittal MRI scan of a CM-I patient showing tonsillar ectopia below the McRae line; **b** T2-weighted mid-sagittal MRI scan of a healthy control

CM-I is characterized by a displacement of cerebellar tonsils through the foramen magnum (> 3–5 mm below McRae line) (Lawrence et al., 2018), which leads to a significant craniocervical junction compression (Meadows et al., 2001; Smith et al., 2013) (see Fig. 1). The diagnosis typically takes place at around 30–40 years old (Avellaneda et al., 2009), but there are also pediatric patients (Massimi et al., 2021). About its etiology, theories suggesting osseus dysplasia are widely accepted (Marin-Padilla & Marin-Padilla, 1981; Meadows et al., 2001), which are also supported by genetic findings (Capra et al., 2019; Markunas et al., 2020; Urbizu et al., 2017). Also, a loss of compliance of the spinal canal in the cervical spine area has been proposed as a cause of CM-I symptoms (García et al., 2022; Labuda et al., 2022).

The heterogeneity among CM-I patients ranges from asymptomatic cases to those with serious medical complications. The most frequent complaints include headaches and cervical pain, in addition to a set of symptoms attributed to craniocervical compression and cerebrospinal fluid (CSF) flow disturbances (i.e., limb weakness, sensory and motor changes) (Avellaneda et al., 2009; Novegno, 2019). Literature about CM-I outcomes has also reported high diversity among patients. Symptomatic patients show a positive trend after surgery, with less percentage of worsening, and those who are asymptomatic present a greater stability of their clinical manifestations and amigdalar ectopia (Arnautovic et al., 2015; Chatrath et al., 2019; Langridge et al., 2017). Despite the progress, the understanding of the natural history of CM-I requires further research of longer clinical and imaging follow-up (Maher, 2020). Usually, the most administered treatment for CM-I patients consists of posterior fossa decompression (PFD) surgery, which has shown favorable results (Mueller & Oro’, 2005; Parker et al., 2013), mainly combined with other techniques such as duraplasty (Chai et al., 2018; Chen et al., 2017; Kumar et al., 2018; Zhao et al., 2016).

While research about clinical manifestations, surgical outcomes, or improvements in medical treatments is still growing, cognitive functioning in CM-I patients has been less explored. Nevertheless, recent studies also highlight the importance of considering the cognitive disturbances in the clinical profile of CM-I patients (Allen et al., 2014; Fischbein et al., 2015; Rogers et al., 2018). Both physical and cognitive deficits affect negatively CM-I patients’ quality of life (Bakim et al., 2013; Meeker et al., 2015), reporting high rates of anxious-depressive symptomatology (Fischbein et al., 2015; Garcia et al., 2019; Mestres et al., 2012). It is therefore meaningful a more comprehensive approach in this pathology, considering physical, psychological, and cognitive status of patients.

At the end of twentieth century, the conceptualization of the cerebellum changed, associating cognitive and affective processes to this structure in addition to the classically associated motor functions (Leiner et al., 1986, 1993; Schmahmann, 2019). Schmahmann and Sherman (1998) proposed the “cerebellar cognitive affective syndrome” (CCAS) to define a set of cognitive disturbances that includes four main areas: executive functioning, spatial cognition, language, and personality. The CCAS has been reported across different cerebellar disorders (Argyropoulos et al., 2020), and it can be assessed through a specific scale (Hoche et al., 2018). Moreover, neuroimaging studies have suggested strong evidence about anatomical and functional connectivity between cerebellum and cortical areas (Buckner et al., 2011; Guell & Schmahmann, 2020; Houston et al., 2021; Stoodley et al., 2012). Particularly, the posterior lobe of cerebellum has been directly related to cognitive functions, while anterior lobe has a main role in motor tasks (Stoodley et al., 2016). Recent research suggests that fiber connection disruptions could affect cognitive performance controlled by certain cortical regions (Habas et al., 2009; Schmahmann, et al., 2019; Stoodley et al., 2012). Houston et al. (2021) showed that intrinsic functional connectivity in CM-I using resting-state fMRI methods showed hypo-connectivity relative to healthy controls for cognitive associations, but hyper-connectivity when associated with pain (i.e., CM-I patients showed hyper-connectivity with cortical areas associated with pain relative to healthy controls).

Rogers et al. (2018) published a systematic review of cognition in CM-I, showing that CM-I patients may experience cognitive dysfunctions, in addition to clinical symptomatology. Likewise, this article shows the paucity of scientific research focused on the cognitive impact of CM-I. Since that publication, new articles have been published that extend our understanding of cognitive dysfunction associated with this disorder. Also, Houston et al. (2022) showed the importance of considering pain effects when considering cognitive effects in CM-I. For example, distraction due to pain can result in cognitive dysfunction in CM-I (e.g., Allen et al., 2018), so it is important to consider whether there are cognitive effects in CM-I that are separate from the distracting effect of pain. The aim of this present study is to conduct a systematic review to update that of Rogers et al. (2018) and Houston et al. (2022), considering adult and pediatric populations, and to point out the significant developments and remaining challenges.

## Method

This study has been conducted considering the PICOS criteria (see Table 2) and following the Preferred Reporting Items for Systematic Reviews and Meta-Analyses (PRISMA) methodology.

**Table 2:** Search strategy according to PICOS criteria (Population; intervention; comparison; outcome; study design)

P	People with Chiari malformation type I diagnosis. No restriction on age, surgery status, culture
I	Any cognitive or neuropsychological assessment
C	Any group comparison or studies without sample comparison
O	Cognitive performance (cross-sectional and longitudinal studies)
S	Empirical studies (any sample size)

### Search Strategy

A systematic review was carried out to identify original empirical studies that assessed cognitive functioning in CM-I patients. We queried the following online databases: MEDLINE, PsychINFO, Pubmed, Cochrane Library, Scopus, and Web of Science. We used a combination of the following keywords: “Chiari malformation” and “cognitive,” “neuropsychological,” “cognitive assessment,” “neuropsychological assessment,” “cognition,” and “neuropsychology.”

The literature search was conducted until February 1, 2023.

### Inclusion and Exclusion Criteria

To be included in the review, studies had to (a) include pediatric or adult participants with a CM-I diagnosis, (b) include cognitive or neuropsychological assessment with standardized tests (one or more determinants of cognitive performance), (c) be published in English or Spanish in a scientific journal, and (d) be empirical studies. For the purposes of this study, both with and without decompressive surgery patients were considered. Patients with comorbid diagnoses were also included if those were related to CM-I disorder (e.g., syringomyelia).

Articles that did not report empirical data were excluded from the review (i.e., review articles, meta-analyses, descriptive observations, expert commentaries). Data from textbooks and conference abstracts were also excluded.

### Data Extraction

**Fig. 2:**
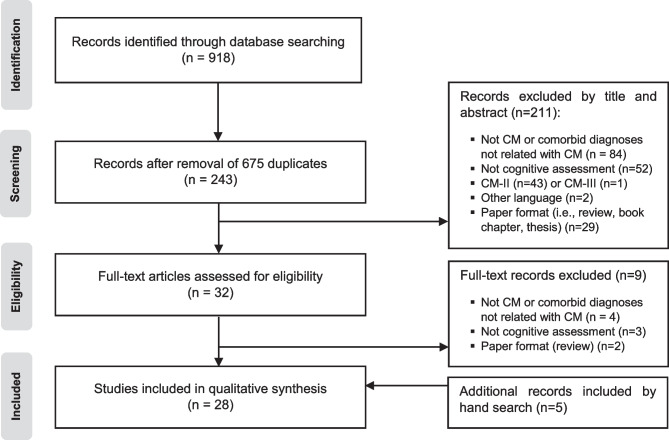
Flowchart of the literature search

The update search yielded 918 studies (M.G.). After eliminating duplicates, 243 were screened by two independent authors (M.G. and M.P.) analyzing the titles and abstracts. After the screening, those potentially suitable (*n* = 32) were fully examined (M.G. and I.A.). A final consensus regarding the eligibility of each article was reached by discussion between both reviewers (M.G. and I.A.). Finally, 23 articles were obtained. First round for data extraction was conducted by one researcher (M.G.) and later, other reviewer (M.P.) assessed the accuracy of the extracted data. Studies that met the established criteria were summarized according to (i) authors, (ii) sample size, (iii) sample characteristics (age and gender), (iv) surgical treatment, (v) clinical signs and symptoms, (vi) neuropsychological tests, (vii) cognitive deficits, and (viii) cerebral imaging data of interest. The reference lists of selected articles were reviewed for possible additional publications and five studies were also obtained by manual searches, resulting in 28 articles included in this systematic review. The flow diagram of identification of relevant studies is shown in Fig. 2.

### Risk of Bias

Risk of bias was evaluated for each included study using the adapted version of the modified Newcastle–Ottawa Scale (Bawor et al., 2014). This scale was used following the background of the previous systematic review about cognition in CM-I (Rogers et al., 2018). It allows to examine quality of evidence of each publication considering different domains: participants selection (selection bias), control confounding (performance bias), statistical methods (detection bias) and outcome measures (information bias). Each item ranges from 0 (high risk) to 3 (low risk). No studies were excluded on the basis of risk of bias.

## Results

### General Overview

Of the initial 918 considered articles, 28 studies met the eligibility criteria for inclusion in this systematic review. Selected articles with adult patients are shown in Table 3 (*n* = 21), and articles with pediatric samples are listed in Table 4 (*n* = 7). From a global view, only two studies reported no deficits in CM-I patients (Almotairi et al., 2020; Klein et al., 2014), whereas the remaining 26 studies reported at least one cognitive domain decreased in CM-I patients. Moreover, the vast majority of selected articles included a sample size below 30 CM-I participants, although five articles exceeded samples sizes of 30 CM-I participants (Allen et al., 2018; García et al., 2018b; Lacy et al., 2016; Lázaro et al., 2018). As regards the latter issue, smaller sample sizes are one of the main limitations of the cognitive studies conducted in CM-I patients (although these studies typically include both CM-I and healthy control samples). It should also be noted that there exists considerable heterogeneity in the methods used in these studies as well as the diversity of cognitive tests and protocols, ranging from screening tasks such as Minimental State Examination (MMSE) (Pearce et al., 2006) to comprehensive batteries such as Wechsler Adult Intelligence Scale (WAIS) or Wechsler Preschool and Primary Scale of Intelligence (WIPPSI) (e.g., García et al., 2018a; Klein et al., 2014; Novegno et al., 2008). Thus, the comparison across different researches is limited, and it is difficult to draw a clear conclusion from them.

**Table 3:** Cognitive studies in adult population diagnosed with CM-I

**Author**	***n***	**Control group**	**Age**	**Gender**	**Surgical treatment**	**Clinical signs and symptoms**	**Neuropsychological tests**	**Cognitive deficits**	**Neuroimaging findings**
Pearce et al. (2006)	1	–	28	1 female	Yes *VSB	Ectopia: non-specified Scoliosis, hydrocephalus, quadriparesis	MMSE Others unspecified	Orientation; attention	N/A
Kumar et al. (2011)	10	10 Matched: age; gender	18–36	3 female 7 male	Non-specified	Ectopia: ≥ 5 mm Headache, dizziness	WAIS: Picture completion, Digit-symbol coding, Picture arrangement, Object assembly, Block design TMT	EF; visuospatial abilities; processing speed	MRI: • Ectopia below the foramen magnum DTI: • Decreased FA and increased MD in putamen, genu, splenium and fornix • Increased MD in cingulum • Increased AD in putamen, thalamus and fornix • Increased RD in fornix and cingulum
Del Casale et al. (2012)	1	–	18	1 male	No	Ectopia: non-specified Vertebral synostosis, basilar impression, modest dilatation of the central canal	RPM Stroop Corsi blocks Verbal fluency (phonological and semantic)	Visuospatial memory; psychomotor retardation; verbal fluency	MRI: • Ectopia below the foramen magnum • Posterior fossa volume and skull base anomalies
Mahgoub et al. (2012)	1	–	53	1 female	Non-specified	Ectopia: non-specified Impaired coordination, vertigo	MMSE MoCA Clock drawing DRS	EF; orientation; recall; attention; abstraction Generalized deficit	MRI: • Arnold-Chiari I malformation
Allen et al. (2014)	24	24 Matched: age; years of education	15–59	22 female 2 male	Yes (*n* = 24) *PFD	Ectopia: non-specified Headache, dizziness, balance issues	RAVLT Stroop Ospan Digity-symbol coding	Response inhibition; generalized slowing	MRI: • Cerebellar herniation below the foramen magnum
Klein et al. (2014)	2	–	a) 19 b) 38	2 male	No	a) Ectopia: 10 mm Headache, vertigo, syncope, muscle spasm b) Ectopia: 6 mm Headaches, muscle spasm	Stanford-Binet 5 RBANS CVLT CPT-II TMT WCST D-KEFS: Torre (only b) DSMB Grooved Pegboard ROCFT TPT WRAT	No significant deficits	MRI: • Cerebellar tonsils herniation below the foramen magnum • Posterior fossa volume anomalies
Allen et al. (2018)	638	102 Not matched	18–64	Non-specified	Mixed Yes (*n* = 341) *PFD	Ectopia: • PFD: 9.53 mm • Non-PFD: 7.01 mm Chronic pain	RAVLT	Immediate recall; attention	MRI: PFD (*n* = 121) and Non-PFD (*n* = 132) • Cerebellar tonsils herniation below the foramen magnum (> 5 mm)
Houston et al. (2018)	19	19 Matched: age; gender; years of education	35.4 ± 11.7	18 female 1 male	Mixed Yes (*n* = 2) *PFD	Ectopia: non-specified	RBANS: Digit-symbol coding, Digit forward Facial emotion perception task (NimStim database)	Emotional regulation; attention; processing speed	EEG and Event-Related potentials: Neurophysiological activity anomalies (consistent with a dysfunctional fronto-parietal attentional network)
García et al. (2018b)	39	39 Matched: age; gender; years of education	45.59 ± 11.9	32 female 7 male	No	Ectopia: 8.51 ± 4.81 Headache, dizziness, muscle pain, muscle weakness, trouble sleeping, syringomyelia (25.6%)	WAIS-IV: Digit backward Zoo map Stroop COWA SCVLT BNT ROCFT SDMT FEEL BFRT Happe’s Stories	EF; verbal fluency, visuospatial abilities, naming ability, verbal memory, processing speed, ToM	N/A
Besteiro and Torres (2018)	20	20 Matched: educational level	38–60	14 female 6 male	No	Ectopia: ≥ 5 mm	Stroop WCST CPT	EF; inhibition; attention	MRI: • Tonsillar herniation below the foramen magnum (> 5 mm)
Lázaro et al. (2018)	51	50 Matched: age; gender; educational level	48.62 ± 10.9	41 female 10 male	Mixed Yes (*n* = 27) *PFD	Ectopia: non-specified Syringomyelia (36.5%), hydrocephalus (4%)	COWA	Verbal fluency (phonetic and semantic)	N/A
García et al. (2018a)	76	76 Matched: age; gender; years of education	PFD: 51.32 ± 9.59 Non-PFD: 45.59 ± 11.9	63 female 13 male	Mixed Yes (*n* = 37) *PFD	Ectopia: PFD: 12.0 ± 7.0 Non-PFD: 8.51 ± 4.81 Headache, dizziness, neck-back pain, muscle pain, muscle weakness, trouble sleeping, subjective cognitive deficits, syringomyelia (40.5%_PFD_/25.6%_Non-PFD_), hydrocephalus (13.5%_PFD_)	WAIS-IV: Digit backward Zoo map Stroop COWA SCVLT BNT ROCFT SDMT FEEL Happe’s Stories	EF; verbal fluency; spatial cognition; naming ability; verbal memory; processing speed; emotional facial recognition; ToM	N/A
Lacy et al. (2019)	18	18 Matched: age; years of education	30 ± 8.62	15 female 3 male	No	Ectopia: non-specified	MMSE RBANS Stroop D-KEFS: Card sorting TMT-B	Generalized deficit; verbal fluency; immediate memory; spatial planning abilities	N/A
Houston et al. (2019)	18	18 Matched: age; gender; years of education	34.4 ± 12.7	17 female 1 male	No	Ectopia 12.4 ± 4.9 mm Headache, neck pain, limb pain, fatigue, dizziness, tinnitus, insomnia, difficulty concentrating, forgetfulness, syringomyelia (22.2%)	RBANS	Attention, immediate and delayed memory	MRI: • Cerebellar tonsils herniation below the foramen magnum. Greater descent correlated with worse delayed memory • Posterior fossa volume anomalies and shorter intracranial heights. Greater osseous areas correlated with better attention
García et al. (2020b)	26	26 Matched: age; gender; years of education	47.15 ± 14.6	22 female 4 male	Mixed Yes (*n* = 8) *PFD	Ectopia: 7.74 ± 3.95 Headache, body pain, muscle weakness, sensory disturbances, dizziness, vertigo, instability, fatigue, visual and hearing alterations, nystagmus, swallowing problems, sphincter problems, sleeping disturbances, cognitive subjective deficits, syringomyelia (26.9%), hydrocephalus (7.7%)	WAIS-IV: Block design, Visual puzzles ROCFT JoLO WAT	Visuospatial abilities (mental imagery)	N/A
García et al. (2020a)	26	26 Matched: age; gender; years of education	47.15 ± 14.6	22 female 4 male	Mixed Yes (*n* = 8) *PFD	Ectopia: 7.74 ± 3.95 Headache, body pain, muscle weakness, sensory disturbances, dizziness, vertigo, instability, fatigue, visual and hearing alterations, nystagmus, swallowing problems, sphincter problems, sleeping disturbances, cognitive subjective deficits, syringomyelia (26.9%), hydrocephalus (7.7%)	Faux Pas test Happé’s Stories Ice-cream Van task FEEL WAT	ToM	N/A
Almotairi et al. (2020)	13	–	37 ± 12	10 female 3 male	Yes (*n* = 13) *PFD	Ectopia: 5–12 mm (46%) 12.1–20 mm (38%) > 20 mm (15%) Headache, weakness, sensory loss, blurred vision, swallowing difficulty, tremor, gait difficulty dysarthria, nausea, syringomyelia (54%)	*n* = 11 (2 missed) WAIS: Digit-symbol coding RAVLT Stroop BVMT-R Grooved Pegboard	No significant deficits *Improvement after surgery: verbal learning, psychomotor and verbal speed, inhibition	N/A
Houston et al. (2020)	18	18 Matched: age; gender; years of education	33.8 ± 11.4	18 female	Mixed Yes (*n* = 2) *PFD	Ectopia 12.5 ± 4.6 mm Headache, neck pain, limb pain, fatigue, dizziness, tinnitus, insomnia, difficulty concentrating, forgetfulness, syringomyelia (22.2%)	RBANS: Digit-symbol coding, Digit forward	Attention; processing speed	DTI: • Increased FA in internal capsule, corpus callosum, stratum, longitudinal fasciculus, middle cerebellar peduncle and corona radiata • Decreased RD in the left anterior corona radiata • Decreased MD in the corpus callosum and left superior longitudinal fasciculus *After pain effect controlling, FA, RD and MD differences were eliminated
Houston et al. (2021)	17	18 Matched: age; years of education	32.5 ± 10.1	16 female 1 male	Mixed Yes (*n* = 2) *PFD	Ectopia 12.6 ± 4.8 mm Headache, neck pain, limb pain, fatigue, dizziness, tinnitus, insomnia, difficulty concentrating, forgetfulness, syringomyelia (22.2%)	RBANS: Digit-symbol coding, Digit forward	Attention	fMRI: • Hyperconnectivity between the PCC and the left globus pallidus, and between the cerebellar lobule VIII and the left postcentral gyrus and vermis IX and the precuneus • Hypoconnectivity between PCP and the right supramarginal gyrus
Seaman et al. (2021)	Pre: 26 Post: 19	–	Pre/post: 18.4–56.2	Pre: 23 female 3 male Post: 19 female	Yes (*n* = 26) *PFD (extradural or intradural)	Ectopia 15.2 ± 4.8 mm Headache, bulbar signs or symptoms, nystagmus, scoliosis, syringomyelia (42%)	WAIS-IV: Similarities, Vocabulary, Block design, Matrix reasoning, Digit span, Symbol search, Digit symbol coding Stroop RAVLT TMT (A-B) COWA WRAT ROCFT WMS: Spatial span JoLO Grooved Pegboard	Visuospatial abilities; visual memory; psychomotor abilities *No differences were found after surgery	N/A
Yilmaz et al. (2022)	29	33 Matched: age; gender educational level	37.6 ± 9.9	24 female 5 male	No	Ectopia: non-specified	MMSE Stroop Kent EGY Porteus Maze NSLT	Generalized cognitive deficit and slowing; EF; response inhibition	N/A

Sociodemographic, clinical, cognitive, and imaging data are reported from CM-I patients, not control groups

*AD* Axial Diffusivity, *BFRT* Benton Facial Recognition Test, *BNT* Boston Naming Test, *BVMT-R* Brief Visuospatial Memory Test-revised, *CPT* Conners` Continuous Performance Test, *CVLT* California Verbal Learning Test, *D-KEFS* Delis-Kaplan Executive Function System, *DRS* Dementia Rating Scale, *DSMB* Dean–Woodcock Sensory Motor Battery, *EF* Executive Functioning, *FA* Fractional Anisotropy, *FEEL* Facially Expressed Emotion Labeling Test, *JoLO* Judgment of Line Orientation Test, *MD* Mean Diffusivity, *MMSE* Minimental State Examination, *MoCA* Montreal Cognitive Assessment, *NSLT* Number Sequencing Learning Test, *Ospan* Operation Span Task, *PFD* Posterior Fossa Decompression, *PCC* Posterior Cingulate Cortex *PCP* Posterior Cerebellar Pathway, *RAVLT* Rey Auditory Verbal Learning Test, *RD* Radial Diffusivity, *RBANS* Repeatable Battery for the Assessment of Neuropsychological Status, *ROCF* Rey-Osterrieth Complex Figure Test, *RPM* Raven’s Progressive Matrices, *SDMT* Symbol Digit Modalities Test, *SPVLT* Spain-Complutense Verbal Learning Test: *TMT* Trail Making Test, *ToM* Theory of Mind, *TPT* Tactile Performance Test, *VSB* Ventriculoperitoneal Shunt Blockade, *WAIS* Wechsler Adult Intelligence Scale, *WAT* Word Accentuation Test, *WCST* Wisconsin Card Sorting Test, *WMS* Wechsler Memory Scale, *WRAT* Wechsler Reading Ability Test

**Table 4:** Cognitive studies in pediatric population diagnosed with CM-I

**Author**	***n***	**Control group**	**Age**	**Gender**	**Surgical treatment**	**Clinical signs and symptoms**	**Neuropsychological tests**	**Cognitive deficits**	**Neuroimaging findings**
Gabrielli et al. (1998)	6	–	4–16	Non-specified	Non-specified	Ectopia: non-specified Mild mental retardation	Brunet-Lezine (< 2 y.o.) Stanford-Binet (2–6 y.o.) WISC-R (> 6 y.o.)	Generalized cognitive deficit (IQ below 70)	MRI: • Chiari-related anatomical signs
Grosso et al. (2001)	9	–	6.4–13.5	5 female 4 male	Non-specified	Ectopia: 10.1 (7.8–15 mm) Speech delay, dysarthria, epileptic episodes, EEG abnormalities, facial dysmophisms, axial hypotonia	WISC BVMT Goodenough	Generalized cognitive deficit (IQ below 50); delayed speech acquisition	MRI: • Thin corpus callosum and wide cavum septum pellucidum (*n* = 3)
Haapanen (2007)	1	–	7	1 male	Non-specified	Ectopia: 10 mm EEG abnormalities	Non-specified	Mild intelectual deficit; delayed speech acquisition; auditory memory; psychomotor retardation	MRI: • Chiari-related anatomical signs
Novegno et al. (2008)	10	–	1–16	Non-specified	Non-specified	Ectopia (4/10): > 10 mm Headache, cervical pain, upper extremity pain, vertigo, seizures, gait instability	WIPPSI WISC-R GMDS Visual Motor and Perceptual Integration ROCFT Gauthier test	Slowed speech, poor fluency, visual attention disturbances, dyspraxia, visual memory	MRI: • Chiari-related anatomical signs
Riva et al. (2011)	2	–	a) 5 b) 15	(a) 1 male (b) 1 female	Yes (*n* = 2) *PDF	Ectopia > 10 mm	GMDS Other non-specified	EF; attention and language abilities (contrasting results) *Improvement after surgery: language (a), attention (b) *Worsening after surgery: attention (a), language (b)	N/A
Lacy et al. (2016)	77	–	6–17	36 femenino 41 masculino	Mixed Yes (*n* = 38) *PFD	Ectopia: non-specified Headache, generalized pain, swallowing and coughing difficulties, gait disturbances, seizures, hydrocephalus (10%), syringomyelia (16%), pseudotumor cerebri (10%)	BRIEF—parent form	EF, working memory, metacognition	N/A
Sari and Ozum (2021)	10	13 Matched: age; gender	12–18	7 female 3 male	No	Ectopia: non-specified Asymptomatic, incidentally detected by MRI	BRIEF—parent form Stroop CPRS-R:S TMT Kent EGY Porteus Maze	Generalized cognitive deficit and slowing; lower IQ	N/A

Sociodemographic, clinical, cognitive, and imaging data are reported from CM-I patients, not control groups

*BRIEF* Behavior Rating Inventory of Executive Functioning, *BVMT* Bender’s Visual-Motor Test, *CPRS-R:S* Conner’s Parent Rating Scale-Revised short form, *GMDS* Griffith Mental Developmental Scale, *IQ* Intelligence Quotient, *PFD* Posterior Fossa Decompression, *ROCFT* Rey-Osterrieth Complex Figure Test, *WISC* Wechsler Intelligence Scale for Children, *WIPPSI* Wechsler Preschool and Primary Scale of Intelligence

Risk of bias and overall quality of evidence analyses showed that sample-related methods need to be improved in further research. The main remaining challenges are focused on larger sample sizes and longitudinal studies (Table 5).

**Table 5:** Quality indicators across reviewed studies

	**Method for selecting sample**	**Methods to control cofounding**		**Statistical methods**		**Methods for measuring outcomes**	
		**Sample size**	**Identification of cofounders**	**Appropiate analyses**	**Missing data**	**Outcome measures**	**Objective assessment**
**CM-I adult population**							
Pearce et al. (2006)	↑	↑	↑	↑	↓	↔	↓
Kumar et al. (2011)	↔	↑	↑	↔	↓	↔	↓
Del Casale et al. (2012)	↑	↑	↑	↑	↓	↔	↓
Mahgoub et al. (2012)	↑	↑	↑	↑	↓	↔	↓
Allen et al. (2014)	↔	↔	↔	↓	↓	↓	↓
Klein et al. (2014)	↑	↑	↔	↑	↓	↔	↓
Allen et al. (2018)	↔	↓	↑	↔	↓	↔	↓
Houston et al. (2018)	↔	↔	↓	↓	↓	↓	↓
García et al. (2018b)	↔	↔	↓	↓	↓	↓	↓
Besteiro and Torres (2018)	↔	↔	↑	↓	↓	↓	↓
Lázaro et al. (2018)	↓	↓	↔	↓	↓	↓	↓
García et al. (2018a)	↓	↓	↓	↓	↓	↓	↓
Lacy et al. (2019)	↔	↔	↔	↔	↓	↓	↓
Houston et al. (2019)	↔	↔	↓	↓	↓	↓	↓
García et al. (2020b)	↔	↔	↓	↓	↓	↓	↓
García et al. (2020a)	↔	↔	↓	↓	↓	↓	↓
Almotairi et al. (2020)	↔	↑	↔	↓	↓	↓	↓
Houston et al. (2020)	↔	↔	↓	↓	↓	↓	↓
Houston et al. (2021)	↔	↔	↓	↓	↓	↓	↓
Seaman et al. (2021)	↔	↔	↓	↓	↓	↓	↓
Yilmaz et al. (2022)	↔	↔	↑	↔	↓	↓	↓
**CM-I pediatric population**							
Gabrielli et al. (1998)	**↑**	**↑**	**↑**	**↑**	**↓**	** ↔ **	**↓**
Grosso et al. (2001)	**↑**	**↑**	**↑**	**↑**	**↓**	** ↔ **	**↓**
Haapanen (2007)	**↑**	**↑**	**↑**	**↑**	**↓**	**↑**	** ↔ **
Novegno et al. (2008)	**↑**	**↑**	**↑**	**↑**	**↓**	** ↔ **	**↓**
Riva et al. (2011)	**↑**	**↑**	**↑**	**↑**	**↓**	** ↔ **	**↓**
Lacy et al. (2016)	** ↔ **	**↓**	**↑**	** ↔ **	**↓**	**↓**	** ↔ **
Sari and Ozum (2021)	** ↔ **	**↑**	**↑**	** ↔ **	**↓**	**↓**	**↓**

Quality indicators have been selected following the adapted version of modified Newcastle–Ottawa scale (Bawor et al., 2014)

High risk of bias: **↑**; Moderate risk of bias: ↔ ; Low risk of bias: **↓**

### Characterization and Cognitive Functioning in Adult Patients

Considering the articles with adult samples, 21 studies met the established criteria. The patient’s age ranged from 15 to 64 years old, and the number of female participants was significantly higher in all group studies. Clinical presentation of CM-I patients was also heterogeneous, ranging from common symptoms such as headache to more severe syndromes such as syringomyelia, which has been referred to in ten studies (Almotairi et al., 2020; García et al., 2018a, b, 2020a, b; Houston et al., 2019; Houston et al., 2020; Houston et al., 2021; Lázaro et al., 2018; Seaman et al., 2021) or hydrocephalus, which has been reported in five articles (García et al., 2018a, 2020a, b, Lázaro et al., 2018; Pearce et al., 2006) (for more details about signs and symptoms, see Table 3).

Of the total of 21 studies, four were case reports (Del Casale et al., 2012; Klein et al., 2014; Mahgoub et al., 2012; Pearce et al., 2006), being three single-case designs (Del Casale et al., 2012; Mahgoub et al., 2012; Pearce et al., 2006). As can be seen in Table 3, only four articles exceeded 30 patients (Allen et al., 2018; García et al., 2018a, b; Lázaro et al., 2018), which points out the lack of large cohort studies as one of the most significant limitation of the literature about cognition in CM-I.

Analyzing the sample design, 15 of a total of 21 articles included a control group (Allen et al., 2014; Allen et al., 2018; Besteiro & Torres, 2018; García et al., 2018a, b, 2020a, b; Houston et al., 2018; Houston et al., 2019; Houston et al., 2020; Houston et al., 2021; Kumar et al., 2011; Lacy et al., 2019; Lázaro et al., 2018; Yilmaz et al., 2022); however, only nine were age-, gender-, and education-matched (García et al., 2018a, b, 2020a, b; Houston et al., 2018; Houston et al., 2019; Houston et al., 2020; Lázaro et al., 2018; Yilmaz et al., 2022). That is another limitation of research examining cognitive functioning in the CM-I population.

There is also considerable diversity in surgical treatment across the studies. Seven of them included CM-I patients without any surgical intervention (Besteiro & Torres, 2018; Del Casale et al., 2012; García et al., 2018b; Houston et al., 2019; Klein et al., 2014; Lacy et al., 2019; Yilmaz et al., 2022), whereas four completed their total samples with undergoing surgery patients (Allen et al., 2014; Almotairi et al., 2020; Pearce et al., 2006; Seaman et al., 2021). Eight contained mixed samples combining patients with and without surgical treatments (Allen et al., 2018; García et al., 2018a, 2020a, b; Houston et al., 2018; Houston et al., 2020; Houston et al., 2021; Lázaro et al., 2018). Two studies did not specify this information (Kumar et al., 2011; Mahgoub et al., 2012). All of the surgical treatments consisted in PFD procedures, except for Pearce et al. (2006) case report, who underwent a ventriculoperitoneal shunt blockade (VSB).

After our literature search, Almotairi et al. (2020) and Seaman et al. (2021) were the only two identified studies that analyzed cognitive functioning in CM-I patients comparing pre- and post-surgical status. In the Almotairi et al.’s (2020) study, 11 patients (two missed from the original sample) were administered neuropsychological assessment both before and after PFD procedure. Before the surgery, they found no significant deficits in cognitive performance but they reported a significant improvement after PFD in the following functions: verbal learning, psychomotor and verbal speed, and inhibitory control. In contrast, Seaman et al. (2021) evaluated 19 CM-I (7 missed from the original sample) through a large battery of neuropsychological tests both before and after PFD, suggesting that CM-I patients had a decreased performance on visuospatial abilities, visual memory, and psychomotor abilities. In addition, contrary to what Amotairi et al.’s (2020) report, no differences were found when comparing results between pre- and post-surgical status (Seaman et al., 2021).

With regard to the adult CM-I patients’ performance across the different cognitive domains, the review suggested a generalized cognitive deficit related to this pathology, apart from physical manifestations such as chronic pain or muscle weakness. A worse performance by CM-I patients has been reported in attention (Allen et al., 2018; Besteiro & Torres, 2018; Houston et al., 2018; Houston et al., 2019; Houston et al., 2020; Houston et al., 2021; Pearce et al., 2006; Mahgoub et al., 2012), orientation (Mahgoub et al., 2012; Pearce et al., 2006), executive functioning (Allen et al., 2014; Besteiro & Torres, 2018; García et al., 2018a, b; Kumar et al., 2011; Mahgoub et al., 2012; Yilmaz et al., 2022), visual (Del Casale et al., 2012; Seaman et al., 2021) and verbal memory (Allen et al., 2018; García et al., 2018a, b; Houston et al., 2019; Lacy et al., 2019; Mahgoub et al., 2012), visuospatial abilities (García et al., 2018a, b, 2020b; Kumar et al., 2011; Lacy et al., 2019; Seaman et al., 2021), processing speed (Allen et al., 2014; Del Casale et al., 2012; García et al., 2018a, b; Houston et al., 2018; Houston et al., 2020; Kumar et al., 2011; Seaman et al., 2021; Yilmaz et al., 2022), verbal fluency (Del Casale et al., 2012; García et al., 2018a, b; Lacy et al., 2019; Lázaro et al., 2018), naming (García et al., 2018a, b), emotional facial recognition (García et al., 2018a; Houston et al., 2018), and social cognition (García et al., 2018a, b, 2020a). Moreover, there are four studies that suggest CM-I patients present a generalized cognitive deficit (Houston et al., 2019; Lacy et al., 2019; Mahgoub et al., 2012; Yilmaz et al., 2022). Nevertheless, as it has been mentioned above, two studies concluded no deficits were present in CM-I patients (Almotairi et al., 2020; Klein et al., 2014).

Seven articles reported a decreased cognitive performance even after controlling for the effects of chronic pain and anxious-depressive symptomatology (Allen et al., 2014; García et al., 2018a, b, 2020a, b; Houston et al., 2019; Houston et al., 2021). In addition, no differences were found between decompressed and non-decompressed CM-I patients in cognitive performance (Allen et al., 2018; García et al., 2018a).

In sum, cognitive functioning appears to be affected in adult CM-I patients, at least some aspects of attention, executive functions, visuospatial abilities, episodic memory, and processing speed, which are the most supported by the literature results. Moreover, this impairment does not seem to be necessarily related to chronic pain or psychiatric symptoms, but there is as yet no clear conclusion regarding the effect of decompressive surgery on cognitive performance.

### Characterization and Cognitive Functioning in Pediatric Patients

The onset age of CM-I is typically around the third decade (e.g., Smith et al., 2013), but it can also be diagnosed in pediatric population, sometimes leading to complex clinical manifestations. In this systematic review, seven studies met the eligibility criteria for the inclusion (Table 4). Patients’ ages ranged from 1 to 18 years old. Regarding gender data, except for Lacy et al.’s (2016) study (41 male vs. 36 female), the number of female participants was superior. Considering signs and symptoms, it was found a high heterogeneity, from asymptomatic patients (Sari & Ozum, 2021) to individuals with common symptomatology related to CM-I such as headache and neck pain (Novegno et al., 2008) or more severe cases with hydrocephalus or syringomyelia (Lacy et al., 2016). Three studies referred to developmental anomalies affecting intellectual abilities (Gabrielli et al., 1998; Grosso et al., 2001; Haapanen, 2007).

Regarding sample size of each study, two studies were case reports (Haapanen, 2007; Riva et al., 2011), of which Riva et al.’s (2011) report was a single-case study. Except for Lacy et al.’s (2016) article, the remaining studies had 10 or less participants. Likewise, only Sari and Ozum (2021) study had an age- and gender-matched control group. In view of these data, methodological limitations are considerable for research examining pediatric subjects with CM-I.

Concerning the effect of surgical treatment, two studies included decompressed subjects in their sample (Lacy et al., 2016; Riva et al., 2011), one included non-operated patients (Sari & Ozum, 2021), and four of them did not specify this information (Gabrielli et al., 1998; Grosso et al., 2001; Haapanen, 2007; Novegno et al., 2008). However, only Riva et al. (2011) analyzed cognitive functioning both before and after surgery. In this study, two case reports were included. The first case was a 5-year-old boy, who was evaluated and showed an improved performance on language abilities but a progressive deterioration of attention after decompressive neurosurgery. The second case was a 15-year-old girl who showed the inverse performance, a worsening in language but an improvement in attention (Riva et al., 2011).

Of the total of seven studies, a generalized cognitive deficit was reported in four of them (Gabrielli et al., 1998; Grosso et al., 2001; Haapanen, 2007; Sari & Ozum., 2021). However, analyzing specific cognitive domains, pediatric CM-I patients showed a decreased performance in the following areas: attention (Novegno et al., 2008; Riva et al., 2011), executive functioning (Lacy et al., 2016; Riva et al., 2011), visual (Novegno et al., 2008) and verbal memory (Haapanen, 2007), processing speed (Haapanen, 2007; Novegno et al., 2008), verbal fluency (Novegno et al., 2008), language (Riva et al., 2011), and metacognition (Lacy et al., 2016). The identified deficits in the Lacy et al.’s (2016) study were collected by parent reports using the Brief Rating Inventory of Executive Functioning (BRIEF). According to their results, CM-I patients had a worse executive functioning regardless their gender, age, or surgical status.

Overall, there is scarce literature about cognitive consequences in pediatric CM-I patients and comparing to adult studies, methodological barriers are more severe. However, there is sufficient evidence to consider cognitive aspects in children with CM-I, especially to prevent delayed acquisition and development of cognitive skills.

### Neuroimaging Data

Analyzing neuroimaging evidence, five studies with adult samples accompanied their neuropsychological findings with brain structure and functional data (Houston et al., 2018, 2019, 2020, 2021; Kumar et al., 2011;). Six studies in adult CM-I patients (Allen et al., 2014; Allen et al., 2018; Besteiro & Torres, 2018; Del Casale et al., 2012; Klein et al., 2014; Mahgoub et al., 2012) and four in pediatric patients (Gabrielli et al., 1998; Grosso et al., 2001; Haapanen, 2007; Novegno et al., 2008) reported information from MRI scans. However, they just indicated Chiari-related anatomical signs such as cerebellar ectopia below the foramen magnum and posterior fossa volumetric anomalies. Lastly, the remaining ten studies with adult patients (Almotairi et al., 2020; García et al., 2018a, b, 2020a, b; Lacy et al., 2019; Lázaro et al., 2018; Pearce et al., 2006; Seaman et al., 2021; Yilmaz et al., 2022) and three with pediatric subjects (Lacy et al., 2016; Riva et al., 2011; Sari & Ozum, 2021) did not include neuroimaging tests in their reports.

To our knowledge, Kumar et al. (2011) were the first study to include neuroimaging tests and their correlation with cognitive performance in CM-I. These authors acquired Diffusion Tensor Imaging (DTI) data from ten CM-I patients and ten healthy controls. When comparing both groups, they found microstructural anomalies in different brain regions. Specifically, they reported the following findings: (a) decreased fractional anisotropy (FA) and increased mean diffusivity (MD) in putamen, genu, splenium, and fornix; (b) increased MD in cingulum; (c) increased axial diffusivity (AD) in putamen, thalamus, and fornix; and (d) increased radial diffusivity (RD) in fornix and cingulum. Moreover, they found some correlations between DTI metrics and cognitive measures such as attention, executive functioning, visuoperceptual abilities, and processing speed. Based on these findings, they suggested a possible deficit in myelination in CM-I patients, leading to an abnormal development of the white matter (Kumar et al., 2011). Following with DTI findings, Houston et al. (2020) reported noteworthy findings according to different parameters: (a) increased FA in the internal capsule, corpus callosum, stratum, longitudinal fasciculus, middle cerebellar peduncle, and corona radiata; (b) decreased RD in the left anterior corona radiata; and (c) decreased MD in the corpus callosum and left superior longitudinal fasciculus. Their study compared 18 CM-I patients and 18 healthy controls; however, when self-reported pain was controlled for, differences between groups in FA, RD, and MD were eliminated.

Houston et al. (2019) correlated brain and cerebellar volumetric measures from MRI scans with cognitive scores obtained through the Repeatable Battery for the Assessment of Neuropsychological Status (RBANS). They assessed 18 CM-I subjects and 18 healthy controls and found that a greater tonsillar descent correlated with worse delayed memory and a greater osseous area correlated with better attention. Apart from cerebellar tonsils herniation below the foramen magnum, they reported posterior fossa volumetric anomalies and shorter intracranial heights in CM-I patients. In a later study, Houston et al. (2021) analyzed the cortico-cerebellar connectivity through a resting-state fMRI technique and reported the following significant abnormalities: (a) hyperconnectivity between the posterior cingulate cortex and the left globus pallidus, and between the cerebellar lobule VIII and the left postcentral gyrus and vermis IX and the precuneus and (b) hypoconnectivity between posterior cerebellar pathway and the right supramarginal gyrus.

Finally, Houston et al. (2018) recorded the neural activity of 19 CM-I patients compared to 19 healthy controls using electroencephalography (EEG). They found neurophysiological activity anomalies consistent with a dysfunctional fronto-parietal attentional network. Although specific data from EEG recording have not been included in more studies of this systematic review, Grosso et al. (2001) and Haapanen (2007) also reported EEG abnormalities in CM-I subjects.

**Fig. 3:**
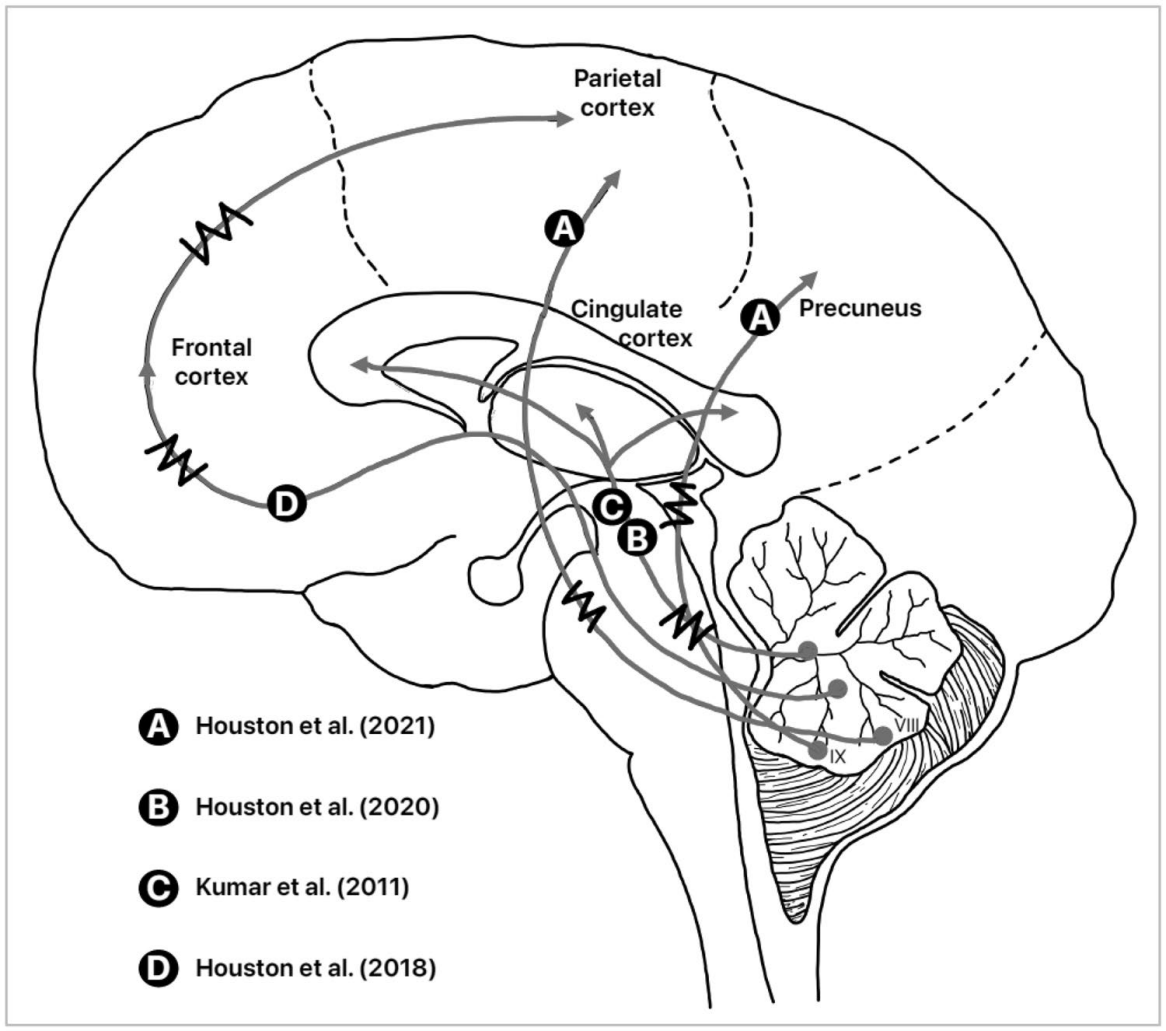
Illustrative summary of the main findings of the studies reviewed

As literature shows, the neuroimaging studies produce useful volumetric and functional information which has suggested the correlation with cognitive measures (see Fig. 3 for an illustrative summary). Nevertheless, and in spite of the latest advances, there is not yet enough evidence to use those parameters as optimal predictors of surgical outcomes, nor as helpful measures to have a deep knowledge about neural mechanisms underlying CM-I disorder.

## Discussion

The initial aim of this systematic review was to update the knowledge of cognitive functioning in CM-I patients. The existing literature has been divided between adult (Table 3) and pediatric studies (Table 4). On the whole, except for two studies (Almotairi et al., 2020; Klein et al., 2014), the remaining works reported at least one cognitive domain decreased in CM-I patients. Although the identified studies were not equally distributed between adult and pediatric samples, both populations have shown cognitive impairments, and it seems to be a partial consensus about the most affected domains, which include attention, executive functioning, visuospatial abilities, episodic memory, and processing speed. However, due to the potential biases and methodological limitations found, it is difficult to draw a common profile of deficits related to CM-I disorder.

Based on the studies we reviewed, we find that there is a notable lack of unanimity in neuropsychological protocols. From large cognitive test batteries to specific tasks, a broad spectrum of cognitive domains has been assessed; however, not all studies included a comprehensive assessment of CM-I patients. This prevents drawing conclusions about certain domains such as social cognition, which has been little studied, or language, remaining less explored in adult population. Moreover, some variables such as chronic pain or anxious-depressive symptomatology have not been controlled for in all studies. Chiari-related pain and psychological symptoms are potential cofounding variables, and the associations between them and cognitive performance should be considered in further research. As we have mentioned before, the vast majority of sample sizes are low and without longitudinal measures; therefore, the interpretation of results and conclusions regarding cognitive functioning of CM-I patients must be done with caution. Moreover, this issue is particularly relevant in pediatric samples with related conditions. In this line, further studies should consider the inclusion of homogeneous matching control groups. Comparing this systematic review with the previous work of Rogers et al. (2018), we can take an optimistic view due to the growing interest in the research about cognitive aspects of CM-I disorder. Since the most recent article included in the Rogers et al.’s (2018) literature review, 15 more works with adult samples have been published and one more with pediatric population. Likewise, some of previous methodological limitations such as small sample size or the inclusion of neuroimaging techniques have been tried to overcome. However, it is still insufficient to obtain a clear cognitive profile related to CM-I disorder.

Considering the most affected cognitive domains that have been indicated in the literature reviewed (attention, executive functioning, visuospatial abilities, memory, and processing speed), there seems to be agreement with the Schmahmann and Sherman’s (1998) syndrome. The CCAS involves executive, visuospatial, and linguistic deficits, which is partly supported by literature when CM-I patients are evaluated. Actually, CM-I disorder has been included as one of the congenital conditions that lead to CCAS (Kraan, 2017). The most supported hypothesis that could explain the cognitive deficits in CM-I patients suggest a dysfunction in cortico-cerebellar circuitry, resulting from structure compression and developmental mechanisms (Steinberg et al., 2020). Neuroimaging studies have identified neural pathways between cerebellum and cortical areas, including motor, parietal, prefrontal, temporal, oculomotor, basal ganglia, and limbic loops (Buckner et al., 2011; D’Angelo & Casali, 2013), which could lead to planning, visual-motor coordination, procedural learning, visuospatial organization, or emotion-related deficits. Habas et al. (2009) studied the involvement of the cerebellum in non-motor functions, suggesting four main connectivity networks: sensorimotor, default mode, executive, and salience network. Similarly, Houston et al. (2021) found group differences in the default mode network activation in CM-I patients compared to controls. To explain the cerebellar role in cognition, the dysmetria of thought theory has been proposed (Guell et al., 2015), which occurs due to a disruption of the cognitive cortico-cerebellar pathways, leading to the CCAS (Schmahmann, 2019). Additionally, the topographic representation of the CCAS has been located in the cerebellar posterior lobe (specifically in VI, VII, and IX hemispheric areas), whereas anterior lobe has motor representation (Schmahmann, 2019). Functional topography studies support the anterior–posterior distinction to locate motor vs. cognitive outcomes (dysmetria of movement vs. dysmetria of thought) following cerebellar lesions (Stoodley et al., 2016). Despite the lack of comprehensive functional neuroimaging studies with CM-I patients and considering the anatomical anomalies in CM-I disorder, it is not unreasonable to think that cognitive deficits were caused by damage to cortico-cerebellar connections.

Research has identified microstructural anomalies in CM-I patients affecting white matter (Abeshaus et al., 2012; Houston et al., 2020; Kumar et al., 2011), and grey matter integrity (Akar et al., 2017; Aydin & Ozoner, 2019). Likewise, both Krishna et al. (2016) and Kurtcan et al. (2018) suggested microstructural alterations in the brainstem region. Volumetric anomalies have also been identified comparing CM-I patients and controls (Biswas et al., 2019). In this systematic review, five studies reported neuroimaging findings, but only three of them found significant correlations between cognitive performance and volumetric (Houston et al., 2019), and functional measures (Houston et al., 2021; Kumar et al., 2011). Regarding the correlation between the magnitude of tonsillar ectopia and cognitive performance, Houston et al. (2019), Grosso et al. (2001) and Novegno et al. (2008) reported a significant finding between those parameters, whereas previous research did not find this association (Crittenden et al., 2017; García et al., 2018b; Stephenson et al., 2017). Moreover, García et al. (2020a) found no correlation between tonsillar ectopia and perceived physical pain reported by CM-I patients. The accumulated evidence about the tissue damaged and volumetric anomalies in CM-I patients may underlie the cognitive dysfunctions reported across the studies. However, this assumption will not be sufficiently validated until an exhaustive study is conducted, including a comprehensive cognitive protocol, longitudinal measures and neuroimaging tests.

A self-report from the CCRC (Conquer Chiari Research Center) revealed that 43.9% of 768 CM-I patients indicated memory problems, 43.8% reported aphasia, 31.6% had problems with decision-making and 29.2% had planning problems (Fischbein et al., 2015). Apart from cognitive complaints, psychological disorders are commonly related to CM-I disorder, such as depression and anxiety, representing in the Fischbein et al.’s (2015) study a percentage of 31.8% and 25.4%, respectively. Higher percentages have been reported in the Garcia et al. (2019) research, in which of the total of 1034 CM-I patients, 44% reported moderate-severe levels of depression, and of the total of 1010 patients, 60% indicated moderate-severe levels of anxiety. These high scores, as well as that reported in this systematic review, reflect that we should not underestimate neurocognitive and psychological symptoms in the clinical presentation of CM-I disorder.

Before the conclusion, it is important to underline the limitations of the studies here reviewed. Although we have set up closed selection criteria to include studies, the vast majority recruited small sample sizes of CM-I patients or presented single-case reports. Likewise, less than half of the studies included a comparative control group, and even less were age-, gender-, and education-matched. Both limitations are more evident in pediatric population. These issues should be addressed in further research, studies with larger sample sizes and more rigorous designs thoroughly considered case–control differences are needed for the Chiari literature body. Moreover, studies administered different neurocognitive measures, and sometimes without controlling for pain and anxious-depressive symptomatology. These factors could limit the comparison of their results and conclusions of cognitive profile in CM-I patients. Chiari-related pain and psychological symptoms are commonly associated with neuroanatomical parameters or specific cognitive deficits; therefore, future studies should consider that relationship. In addition, some patients were also diagnosed with hydrocephalus, making the attribution of cognitive deficits to the CM-I less clear. There are few rigorous studies that compare the effect of decompressive surgery on cognitive performance but these had conflicting conclusions. In addition, few studies included neuroimaging techniques that support cognitive findings analyzing the underlying neural mechanisms. Lastly, there are no longitudinal studies that allow us to know the course of cognitive deficits in CM-I disorder.

## Conclusion

The literature here reviewed seems to yield a partial consensus about the cognitive deficits present in CM-I. Patients mainly showed a lower performance in attention, executive functioning, visuospatial abilities, episodic memory, and processing speed. However, these results require careful interpretation due to the aforementioned methodological limitations of the studies. In addition, although the research of cognitive profile of CM-I has gained increasing attention, the effect of neurosurgery and psychological symptomatology on cognitive performance remains little explored. The quality of life of CM-I patients could be decreased due to the impact of these symptoms on their daily lives, thus, clinicians should include the cognitive assessment in their diagnostic procedures. Researchers should focus on longitudinal designs with larger sample sizes and comprehensive neuropsychological protocols, including neuroimaging data, which is an urgent challenge. In summary, further research is needed to determine a well-defined cognitive profile related to CM-I, favoring a multidisciplinary approach of this disorder.

## Data Availability

Not applicable.
